# Adenovirus Dodecahedron, as a Drug Delivery Vector

**DOI:** 10.1371/journal.pone.0005569

**Published:** 2009-05-15

**Authors:** Monika Zochowska, Agnieszka Paca, Guy Schoehn, Jean-Pierre Andrieu, Jadwiga Chroboczek, Bernard Dublet, Ewa Szolajska

**Affiliations:** 1 Institute of Biochemistry and Biophysics, Polish Academy of Sciences, Warsaw, Poland; 2 Institut de Virologie Moléculaire et Structurale, FRE 2854 CNRS-UJF, Grenoble, France; 3 Institut de Biologie Structurale JP Ebel, CEA, CNRS, UJF, Grenoble, France; Mizoram University, India

## Abstract

**Background:**

Bleomycin (BLM) is an anticancer antibiotic used in many cancer regimens. Its utility is limited by systemic toxicity and dose-dependent pneumonitis able to progress to lung fibrosis. The latter can affect up to nearly 50% of the total patient population, out of which 3% will die. We propose to improve BLM delivery by tethering it to an efficient delivery vector. Adenovirus (Ad) dodecahedron base (DB) is a particulate vector composed of 12 copies of a pentameric viral protein responsible for virus penetration. The vector efficiently penetrates the plasma membrane, is liberated in the cytoplasm and has a propensity to concentrate around the nucleus; up to 300000 particles can be observed in one cell *in vitro*.

**Principal Findings:**

Dodecahedron (Dd) structure is preserved at up to about 50°C at pH 7–8 and during dialysis, freezing and drying in the speed-vac in the presence of 150 mM ammonium sulfate, as well as during lyophilization in the presence of cryoprotectants. The vector is also stable in human serum for 2 h at 37°C. We prepared a Dd-BLM conjugate which upon penetration induced death of transformed cells. Similarly to free bleomycin, Dd-BLM caused dsDNA breaks. Significantly, effective cytotoxic concentration of BLM delivered with Dd was 100 times lower than that of free bleomycin.

**Conclusions/Significance:**

Stability studies show that Dds can be conveniently stored and transported, and can potentially be used for therapeutic purposes under various climates. Successful BLM delivery by Ad Dds demonstrates that the use of virus like particle (VLP) results in significantly improved drug bioavailability. These experiments open new vistas for delivery of non-permeant labile drugs.

## Introduction

Adenovirus (Ad) penton, a non-covalent complex of two oligomeric proteins, fiber and penton base, localized at 12 vertices of the icosahedral virion is responsible for virus entry. The trimeric antenna-like fiber protein attaches the virus to the primary receptor on the host cell plasma membrane while the pentameric penton base, through interaction of its Arg-Gly-Asp containing (RGD) motif with host cell αv integrins, is involved in virus endocytosis. In certain adenovirus serotypes pentons self-assemble into a virus-like particle (VLP) called the dodecahedron (Dd) that is built of 12 pentons, thereby comprising 12 penton bases and 12 fibers [1]. We expressed the penton components of adenovirus serotype 3 in the baculovirus system, which resulted in spontaneous formation of Ad3 dodecahedra in insect cells [2]. In addition, expression under similar conditions of penton base (Pb) alone led to formation of a dodecahedric particle made of 12 bases (dodecahedron base, DB), which demonstrated that dodecahedron is assembled independently of the fiber protein through interaction of only the Pbs. This paper describes the properties of DB, for which both terms, the dodecahedron (Dd) and DB will be used. The term dodecahedron (Dd) will be used throughout this work to describe Ad DB.

Working with the recombinant Dd of serotype 3 (Ad3), our group has shown that Dd components that are responsible for adenovirus cell entry retain these functions in the virus-like particle, which exhibits very efficient internalization and has a propensity to concentrate around the cell nucleus [2]. Since the recombinant wild type Dd harbors no genetic information, it is a safe alternative to adenovirus in gene transfer. In addition, we have recently shown that Dd enters the cell through a heparan sulfate pathway, not used by the virus of origin [3]. This allows Dd to penetrate a wider range of cells than can the adenovirus, the most efficient vector known to date. Finally, studies in our group have demonstrated that Dd is able to translocate DNA to cells, resulting in gene expression [2], as well as to transfer proteins directly, with the transferred protein remaining active inside the transduced cells [4].

To date, VLPs have been produced for more than 30 different viruses (for review see Noad and Roy [5]). This includes viruses with single or multiple capsid proteins as well as those with and without lipid envelopes. Adenovirus is a non-enveloped virus with a dsDNA genome, and among this type of viruses the VLPs have been described for various human papillomavirus (HPV) serotypes, polyomavirus simian virus serotype 40 (SV40), and John Cunningham virus (JC). However, usually these VLPs retain the virions' size and mimic their organization, whereas adenovirus Dd is forming VLPs much smaller than the virion itself and with different intra-particle interactions. Therefore, data on Dd stability cannot be inferred from those obtained for Ad virions (for example, those described by Rexroad and coworkers [6]).

In order to employ the remarkable cell entry properties of Dd for drug delivery, we needed data concerning the vectors' stability. Hence, the stability of Dd was studied under various conditions of salt concentration, pH and temperature. We optimized conditions for vector stability and storage, and analyzed its integrity in cell culture and in human serum. Finally, we used Dd as a vector for delivery of the lipophilic, nonpermeant and labile anticancer antibiotic bleomycin (BLM), which resulted in significant increase in BLM bioavailability.

## Results

### I. DB expression and purification

Ad penton base polypeptides upon expression in human and insect cells oligomerize into pentamers. For Ad3, the majority of pentameric Pbs assemble into symmetrical particles called dodecahedra base (or dodecahedra), of 3.6 MDa, which are made up of 12 pentameric Pbs. In the first step of purification from insect cells the dodecahedric VLPs were isolated from 15–40% sucrose gradient, where they sedimented in 30–40% sucrose, with free pentameric Pbs recovered from lighter sucrose [2].

**Figure 1:**
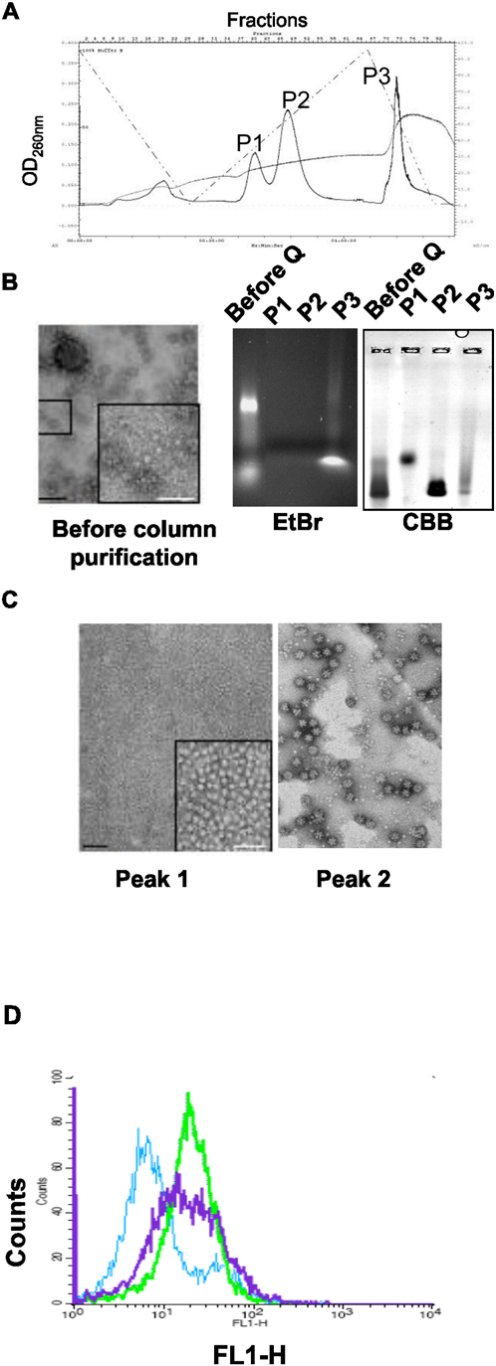
Purification of recombinant Ad3 DB expressed in the baculovirus system. (A) Dodecahedra initially purified on sucrose density gradient were fractionated on a Q-Sepharose column in 20 mM Tris buffer, pH 7.5, using NaCl gradient. (B) Analysis of purified Dds. Left panel - negative stain electron microscopy (EM) of Dds purified on sucrose density gradient. Middle and right panels - non-denaturing agarose gel electrophoresis of fractions recovered from the Q-Sepharose column with detection with ethidium bromide (EtBr, middle panel) followed by Commassie Brilliant Blue (CBB) staining (right panel). (C) Negative stain EM showing free pentameric bases recovered in peak 1 (left panel) and complete dodecahedra in peak 2 (right panel) of the Q-Sepharose column (P1 and P2 in A). Scale bar equals 100 nm. (D) Flow cytometry analysis of HeLa cells transduced with Dd (see Material and Methods). Sucrose density gradient-purified Dds – purple curve, Q-Sepharose-purified Dds - green curve. Blue curve shows the antibody background in the absence of Dd.

Since sucrose fractions containing particles were contaminated with cellular proteins and nucleic acids (Fig. 1A, left panel), final Dds purification was achieved by ion exchange chromatography on a Q-Sepharose column. Pbs containing material was eluted in two peaks: at 280 mM and 380 mM NaCl (P1 and P2 in Fig. 1A), while the third peak observed at 630 mM NaCl contained insect cell DNA (see Fig. 1B, middle panel). Proteins in peaks 1 and 2 migrated with different velocity on native agarose gel (Fig. 1B, right panel). Negative stain electron microscopy (EM) analysis showed non-assembled free base pentamers in peak 1 (Fig. 1C, left panel), whereas the material in peak 2 consisted of dodecahedra bases (Fig. 1C, right panel). It is possible that in sucrose density gradient some Pbs sedimented with the bulk of DBs due to their assembly into larger structures on cellular DNA. Cell penetration assay showed that gradient-purified preparation contained mixed material of varying penetration ability, while Dds obtained from the Q-Sepharose column contained more uniformly penetrating particles (Fig. 1D).

Up to 25% of Dds obtained after sucrose density gradient did not bind to the Q-Sepharose column. Although both pools of unattached and column-purified Dds were able to penetrate cells in culture to a similar extent, the former was unstable upon storage, probably due to proteolysis. DB expression in the baculovirus system was remarkably high, reaching 1 mg of purified particles per about 8×10^6^ insect cells. N-terminal analysis of numerous batches of purified DB showed that it consists of a mixture of N-terminally truncated penton base polypeptides. Cleavage at glycine 9 accounted for approximately 42% of free N-termini, while that at Ala 38, Pro 230 and Val 317 for 13, 7 and 7%, respectively. Interpolating from the data on atomic structure of Ad2 dodecahedron [7], it appears that Val 317 is located in the flexible RGD loop whereas Pro 230 is in an exposed loop near the top of the molecule. The RGD loop is involved in Pb interaction with integrins during adenovirus cell entry, while the second loop is involved in fiber binding and in interactions with the adjacent Pb monomer [7]. It is conceivable that a proteolytic attack on these two exposed loops results in a small portion of dodecahedra being impaired in their penetration ability. Similar proteolysis was observed for simultaneously expressed Ad3 Pb. Our attempts to inhibit proteolysis during the purification process were unsuccessful because the Pb had already been cleaved during expression in insect cells (results not shown).

### II. Studies on DB stability

VLP of SV40 is stabilized by calcium ions and the disulfide bond at Cys 9 prevents SV40 VLPs dissociation, probably by increasing binding of calcium ion [8]. Similarly, the disulfide bonds play an important role in maintaining the integrity of JC virus VLPs by protecting calcium ions from chelation [9]. In contrast, disulfide bonds alone stabilize HPV11, and cation chelation does not affect the stability of HPV11 VLPs [10]. Not much is known about factors involved in Ad3 Dd stability, but preliminary data obtained for Ad3 DB crystals at 3.8 Å resolution showed a metal ion that is almost certainly calcium in the region of contact between subunits (personal communication, Dr S. Cusack). This led us to test the effect of metal-ion chelators on Dd stability. However, Dd retained its structure in the presence of 100 mM EDTA or EGTA (results not shown), suggesting that divalent ions are not involved in maintaining its integrity.

We tested the stability and solubility of purified DB particles over a pH range from 4 to 10.9. For this purpose ion exchange-purified Dds were dialyzed overnight at 4°C against various buffers and subsequently incubated at 30 or 37°C. Following incubation, some preparations were subjected to centrifugation to separate the soluble fraction from the aggregated particles. The assembly status of proteins in the supernatants was analyzed by native agarose gel electrophoresis.

**Figure 2:**
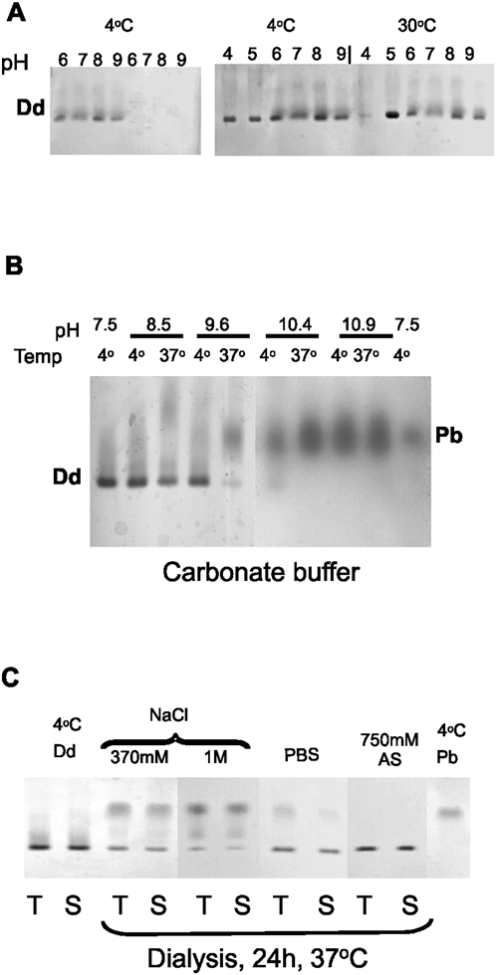
Dd stability under different conditions of temperature, pH and ionic strength, analyzed by native gel electrophoresis. Purified Dds were dialyzed overnight at 4°C (A, B) or for 24 h at 37°C (C) against different buffers at the indicated pH. (A) Effect of pH on Dd solubility. Dd was dialyzed against the following 50 mM buffers: MES, pH 6; Hepes, pH 7; Tris, pH 8 and CAPS, pH 9. Left panel: samples were prepared in duplicates and after dialysis the second batch was centrifuged at 13000 rpm for 30 min. The first four lanes contain the dialyzed samples; the next lanes contain the supernatants after centrifugation. Right panel: samples were dialyzed against the same buffers as described for the left panel and also against citric acid, pH 4, and acetic acid, pH 5, but all buffers contained 150 mM NaCl. Samples were prepared in duplicates and one batch was kept at 4°C while the second one was incubated for 20 min at 30°C. Soluble proteins contained in the supernatants obtained by 30 min centrifugation at 13000 rpm were applied on agarose gel. (B) Dd stability in carbonate buffer. Carbonate buffer (100 mM) was prepared at the indicated pH and used for Dds dialysis. Some samples were incubated at 37°C for 20 min. All samples were centrifuged as above and the supernatants were electrophoresed on native agarose gel. First and last lanes, control Dd and Pb preparations, respectively. (C) Effect of ionic conditions on Dd thermal stability. Dds were dialyzed for 24 h at 37°C against the purification buffer containing NaCl or (NH_4_)_2_SO_4_ (AS) at indicated concentrations or against PBS, pH 7.5. Non-treated samples (T for total) or supernatants after centrifugation at 13000 rpm for 30 min (S) were analyzed on the agarose gel. The first two lanes contain control Dd while the last lane contains Pb preparations, all in purification buffer.

DB particles were readily soluble at 4°C in all the buffers used, but remained in solution only in the presence of 150 mM NaCl (Fig. 2A, four leftside lanes). Without NaCl DB remained in solution in a rather unstable manner, disappearing from the supernatants and pelleting upon centrifugation (Fig. 2A, left panel, four rightside lanes). Clearly, NaCl at a physiological concentration protects DB from precipitation. A protective effect of NaCl was observed also during incubation at 30°C (Fig. 2A, right panel).

For pH values above 8 we used 100 mM sodium carbonate buffer, which up to pH 10.9 did not cause visible protein aggregation, even during incubation at 37°C. However, at pH 10.4 Dds dissociated to free pentameric Pbs, and an increase in temperature to 37°C (30 min incubation) resulted in nearly total DB dissociation already at pH 9.6 (Fig. 2B).

To probe Dd stability upon prolonged temperature treatment, we performed 24 h dialysis at 37°C against buffers of different ionic strength (Fig. 2C). The presence of 370 mM or 1 M NaCl in the purification buffer was sufficient to prevent protein aggregation, but dissociation to free pentameric Pbs was observed under conditions of prolonged temperature treatment. However, the presence of ammonium sulfate significantly stabilized Dd, preventing its dissociation to free pentameric Pbs (Fig. 2C, lanes AS).

In order to apply a more general approach to the question of Dd thermal denaturation, we used dynamic light scattering technique (DLS), which allows monitoring of protein denaturation or unfolding. When denaturation occurs, the size of the protein is increased to a value consistent with a random coil polymer of the same mass. In the absence of aggregation-prohibiting agents, interpolymer hydrophobic interactions can quickly lead to non-specific aggregation of the denatured polypeptide chains. The protein melting point temperature (T_m_), obtained during DLS analysis is indicative of the thermal stability of a protein. A positive shift in T_m_ indicates stabilization of the protein by an increase in structural order and a reduction in conformational flexibility, while a negative shift in T_m_ indicates destabilization. The change in mean particle size that accompanies DB denaturation was measured at different pH and ionic conditions, applying a thermal gradient from 12 to 65°C.

**Figure 3:**
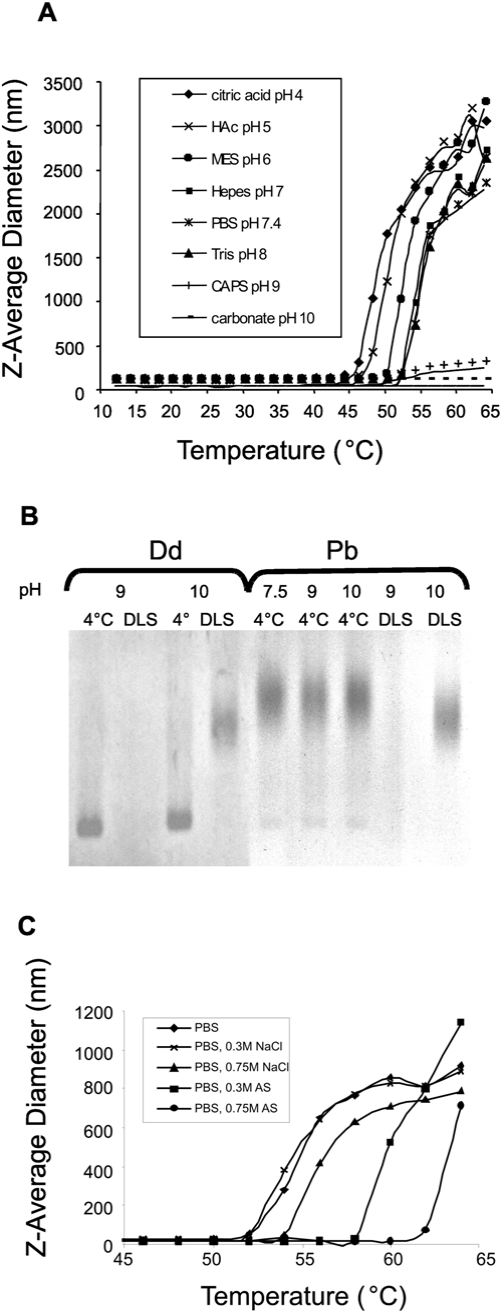
Thermal Dd stability as a function of pH and ionic strength. (A) Dd was analyzed by dynamic light scattering (DLS) at different pH in the presence of 150 mM NaCl as a function of temperature, as described in Material and Methods. (B) Native gel analysis of Dds and Pbs, in CAPS pH 9, and carbonate buffer, pH 10. Some samples were subjected to temperature treatment imitating DLS temperature gradient (marked DLS). (C) DLS analysis carried out on Dds in PBS under different ionic strength conditions. Mean values of three apparatus readings are shown.

Fig. 3A shows the temperature dependent Z-average diameter measurements for DB. At temperatures up to 40°C the particle size was constant at pH 4–9, suggesting a stable structure. Above this temperature the particle size increased exponentially with temperature, indicating the presence of denatured aggregates. Dd denaturation/aggregation started at approximately 10°C lower temperature at pH 4–5 than at pH 7–8. At pH 9 (CAPS) and 10 (carbonate buffer) only small changes in particle size were observed, in particular at pH 10. Native gel analysis of Dd treated with the same temperature gradient as the one applied during DLS analysis (Fig. 3B) showed that while at pH 10 Dd dissociates to free bases, at pH 9 (CAPS buffer) the protein disappears, suggesting aggregate formation. Similar DLS-imitating temperature treatment of free pentameric bases confirmed aggregation of this protein at pH 9 (Fig. 3B, lanes Pb/DLS). Of note, CAPS is an organic buffer that may have a propensity to interact with hydrophobic patches on the protein surface, which can induce protein aggregation. It seems that a temperature increase at pH 4–8 led to rapid Dd aggregation, while at pH 9 Dd first dissociated to free pentameric bases which then underwent aggregation. Addition of 750 mM NaCl to PBS caused a positive shift in the T_m_ of Dd suggesting structure stabilization (Fig. 3C). But the most significant increase in T_m_ values was caused by addition of ammonium sulfate (positive shift of about 12°C, Fig. 3C), which confirmed the stabilizing effect of this salt observed during Dd dialysis at 37°C (Fig. 2C).

### III. Prolonged dodecahedron stability in cultured cells and in human serum *in vitro*

**Figure 4:**
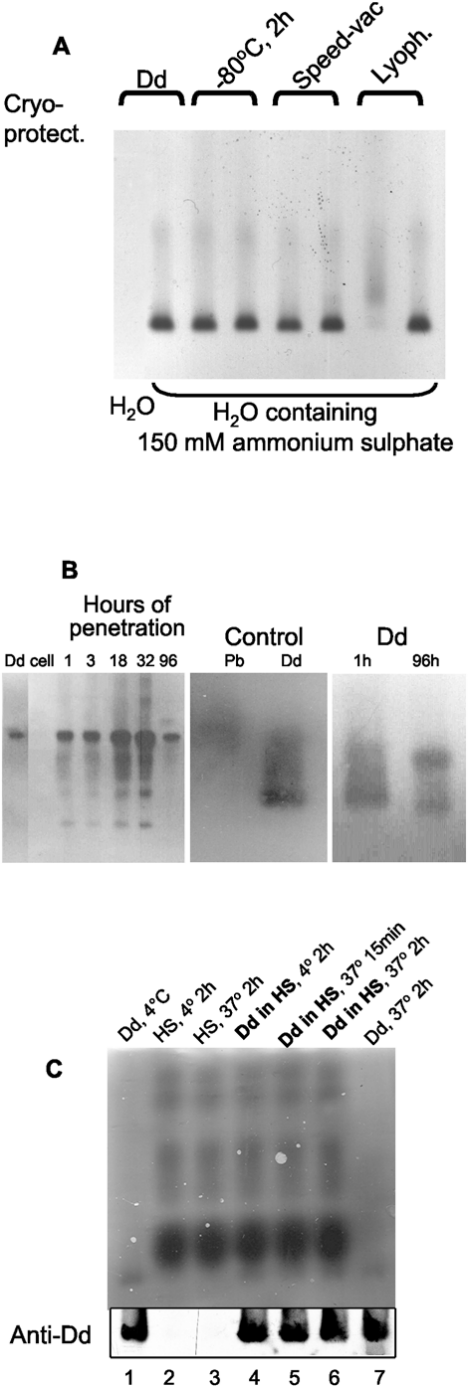
Dd stability upon lyophilization, inside HeLa cells and in human serum. (A) Purified Dds were dialyzed overnight at 4°C against water or 150 mM (NH_4_)_2_SO_4_ in water. Mannitol (0.4%) and sucrose (0.4%) were added to samples marked „Cryoprotect. +”. Dd samples were frozen at −80°C, dried in speed-vac or lyophilized (marked Lyoph. +). Dry samples were reconstituted in the starting volume of water. All preparations were centrifuged for 30 min at 13000 rpm and the supernatants were applied onto native agarose gel. (B) Stability of Dd after application to HeLa cells. Purified Dds (2 µg in 100 µl) were applied to 2×10^4^ portions of HeLa cells. After indicated periods of penetration cell lysates were analyzed on SDS-PAGE (left panel) or on native agarose gel (two right panels). Control Dd samples contained 30 ng protein, while control Pb sample contained 10 ng protein. (C) Stability of Dd upon incubation in human serum. Samples of Dd concentrated by ultrafiltration in Microcon unit (Millipore) (5 µg each) were incubated in human serum (HS) at 4°C for 2 h (lane 4) and at 37°C for 15 min or for 2 h (lanes 5 and 6, respectively). Samples were resolved by native agarose gel electrophoresis and analyzed by Western blot performed with anti-Dd serum. The upper part shows CBB stained gel with proteins remaining after transfer, and the lower part the developed Western blot. Lanes 1 and 7 show Dd non treated or incubated for 2 h at 37°C, respectively, in the absence of serum. Lanes 2 and 3 show human serum after 2 h incubation at 4 and 37°C, respectively. Dd samples incubated with the serum are denoted in bold.

In order to be able to store the vector we tested the effect of freezing and thawing on Dd integrity. After dialysis to water Dd aggregated even in the presence of the cryoprotectants, which was to be expected as it precipitates in the absence of salt (see Fig. 2A). Dd survived freezing at −80°C in 150 mM ammonium sulfate, and then subsequent thawing at room temperature. Dd structure was preserved upon dialysis, freezing and drying in the speed-vac in the presence of 150 mM ammonium sulfate (Fig. 4A). Although during lyophilization of Dd in 150 mM aqueous ammonium sulfate Dd integrity was not maintained, it was preserved in the presence of the cryoprotectants such as 0.4% sucrose and 0.4% mannitol (Fig. 4A, compare the last two lanes).

Intracellular vector survival was tested by Western blotting performed on HeLa cell extracts, which were prepared at different periods following Dd application and resolved on native agarose gels. The amount of intracellular Dd increased significantly up to 32 h post transduction, with noticeable Pb proteolysis resulting in only a fraction of intracellular Pb remaining after 4 days (Fig. 4B, left panel). Native agarose gel analysis showed that 96 h post penetration the majority of intracellular material was running between bona fide Dd and Pb (Fig 4B, right panel, marked with a dot), suggesting the removal of external loops from Dd, albeit with some degree of particle integrity remaining. It is relevant that the anti-Dd antibody recognized proteolyzed Dd forms better than the original ones on native Western blots.

We also tested the integrity of Dd under conditions imitating its potential situation *in vivo*. In freshly prepared human serum the vector seemed to be stable for at least 2 h at 37°C (Fig. 4C).

### IV. Bleomycin delivery by Dd

Bleomycin is a family of metal-binding glycopeptide antibiotics with antimicrobial, antiviral and antitumor properties [11]. BLM is an extremely cytotoxic agent once inside the cell nucleus, where it cleaves the DNA [12], [13]. However, BLM cytotoxicity is limited by its inability to freely diffuse through membranes [14] and by its cleavage by intracellular proteases [15]. In consequence, high doses of BLM have to be used in therapeutic treatment, which in turn leads to serious side effects like non-specific cytotoxicity, including acute lung fibrosis [16].

**Figure 5:**
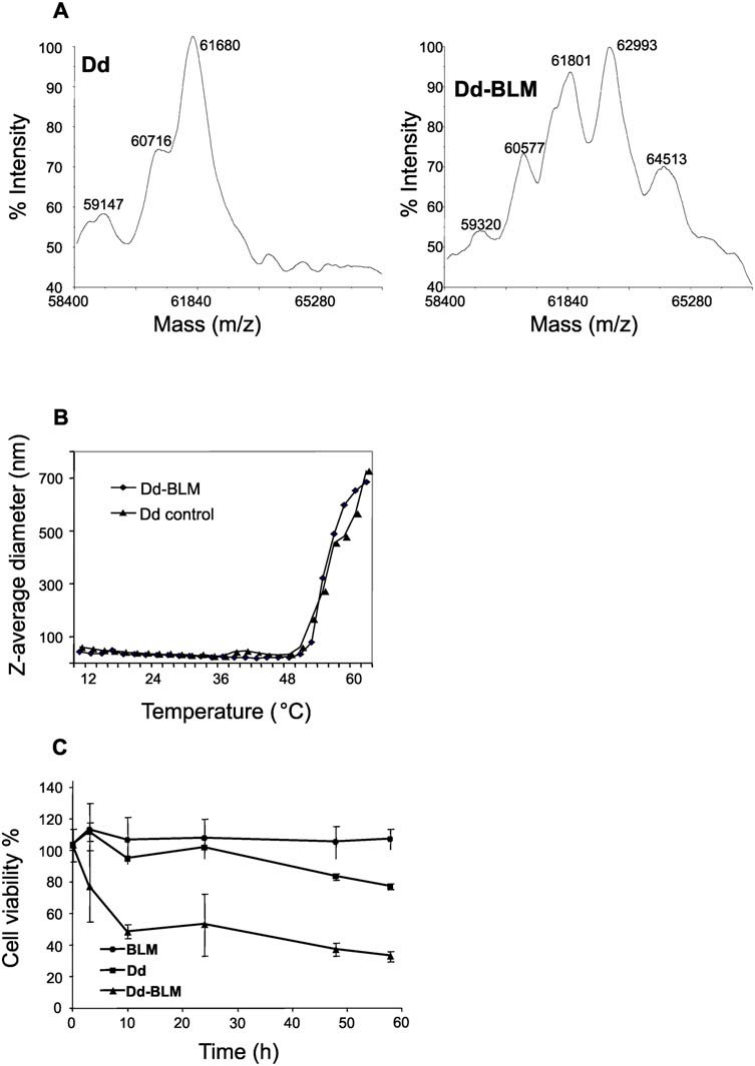
Cell toxicity of bleomycin (BLM) delivered with the aid of Dd. BLM was chemically attached to Dds as described in Material and Methods. (A) Characterization of Dd-BLM conjugate by mass spectrometry analysis (Maldi). (B) DLS analysis of the BLM conjugate. (C) MTT assay of cell toxicity. HeLa cells were treated with free BLM (0.13 µM), Dd (1 µg) and Dd-BLM (1 µg delivering 0.08 µM BLM), as described in Material and Methods.

We attached BLM to Dd chemically, which resulted in a Dd-BLM conjugate with each penton base monomer carrying between 0 and two BLM molecules (BLM molecular mass is 1400), with a clear predominance of the species with one BLM moiety attached, as shown by mass spectroscopy analysis (Fig. 5A). These data suggest that one Dd particle carries on the average 60 BLM molecules. The DLS analysis showed the melting profile of the conjugate to be quite similar to that of untreated Dd (Fig. 5B). Preliminary experiments with cytotoxic concentrations of free BLM revealed that the antibiotic must remain in the culture medium all the time; its removal 3 h after application did not result in cell deaths. Since the amount of intracellular Dd increased significantly up to 32 h post transduction (Fig. 4B, left panel), we analysed the kinetics of cell survival with Dd-BLM conjugate, which showed that at 14 h application about 40% of cells survived while at 48 h application the survival was below 20% cells (results not shown).Therefore, both, BLM and Dd-BLM (as well as Dd alone) were left in the medium throughout the experiment.

The Dd-BLM preparations containing on average 0.08 µM BLM showed significant cytotoxic activity while application of comparable amounts of BLM in the free form did not affect cell growth (Fig. 5C). A comparable level of cell death was observed when at least 8 µM solution of free BLM was used (results not shown).

**Figure 6:**
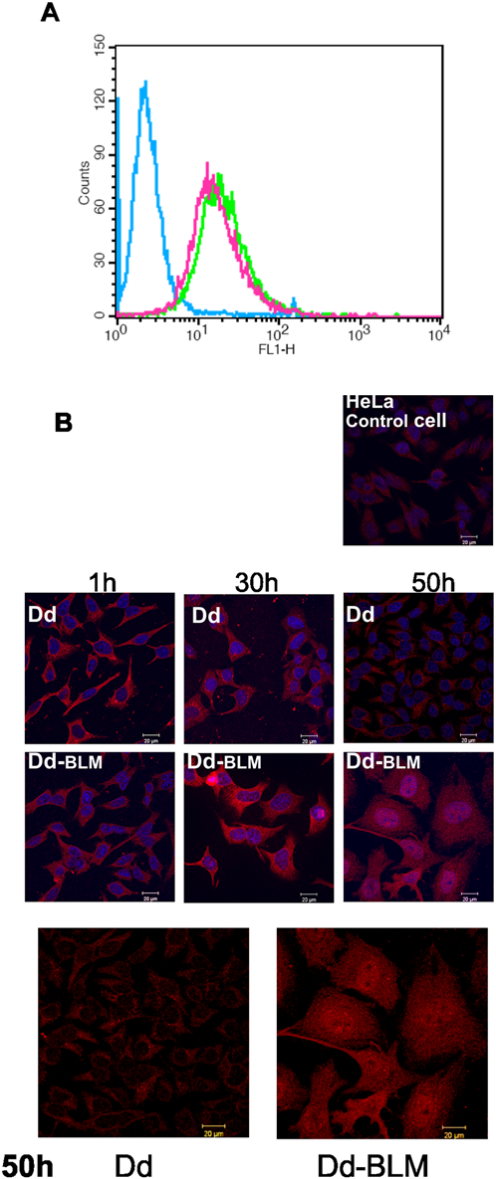
Effect of Dd and Dd-BLM conjugate on HeLa cells. (A) Flow cytometry analysis of Dd (green curve) and Dd-BLM (pink curve) cell entry. Cells were treated with appropriate vector for 1 h at 37°C as described in Material and Methods. The blue curve shows the antibody background in the absence of Dd. (B) Cells were treated with 1 µg Dd or Dd-BLM for indicated times and analyzed with anti-Dd serum (in red) by confocal microscopy, as described in Material and Methods. Nuclei were stained blue with DAPI. Last row shows the 50 h-treatment without nuclear staining. Scale bar equals 20 µm.

The next step involved observation by confocal microscopy. Fascan analysis showed that the entry potential of Dd is not affected by the chemical treatment during conjugate preparation as both free vector particles and the Dd-BML conjugate had similar entry capacity (Fig. 6A). Since the anti-BLM serum appropriate for confocal microscopy is not available, the presence of Dd-BLM conjugate was demonstrated using the anti-Dd antibody. Remarkably, one hour after Dd application all cells were found to be transduced with Dd, whose presence was clearly seen in the cytoplasm of each cell (Fig. 6, 1 h, Dd). At 50 h post application with free Dd, the amount of intracellular Dd significantly diminished with the red signal still limited to the cytoplasm (Fig. 6, 50 h, Dd), suggesting destruction of free Dds and their removal from cells. A 1 h post application cells treated with Dd-BLM exhibited similar image as cells treated with Dd alone at this timepoint (Fig. 6, 1 h). However, at 30 h post application, cells treated with Dd-BLM conjugate appeared somewhat bigger (Fig. 6, 30 h, Dd-BLM). Finally, at 50 h post application Dd-BLM caused the appearance of giant cells with intense red Dd signal in the entire cell, suggesting a collapse of the nuclear membrane (Fig. 6, compare 50 h with Dd versus Dd-BLM, last row, nuclear stain removed).

**Figure 7:**
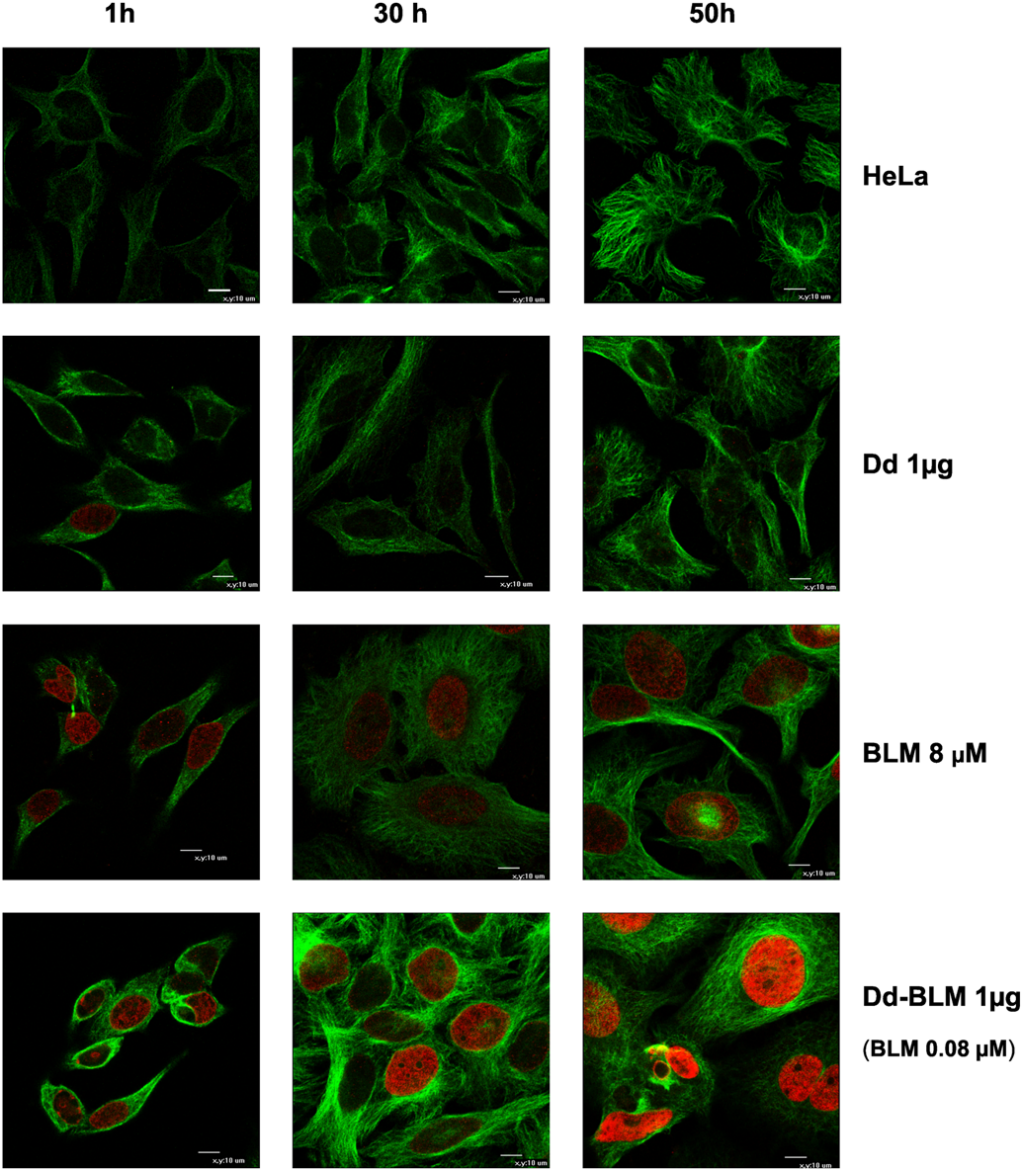
Kinetics of ds DNA breaks as jugded by induction of γ-H2AX foci. HeLa cells were treated either with Dd, with free BLM or Dd-BLM for indicated periods and analyzed with anti-γ-H2AX Ab (in red) and with anti-tubulin Ab (in green) by confocal microscopy, as described in Material and Methods. Scale bar equals 10 µm.

The mechanism of the cytotoxic activity of BLM is based on host DNA cleavage [14]. One of the well-characterized DNA-damage-responsive chromatin modification events is the phosphorylation of the C-terminal tail of histone H2A or the H2AX variant in higher eukaryotes [17]. Therefore, we used the antibody recognizing the phosphorylated form of the H2AX histone for monitoring DNA damage in HeLa cells. Control as well as Dd-treated cells did not show DNA damage as judged by nearly total absence of cells stained red with anti-γ-H2AX (Fig. 7, rows of HeLa and Dd). Similarly, no DNA damage was observed upon application of free BLM up to aproximately 8 µM concentration (not shown). However, at 8 µM free BLM there was significant DNA cleavage at all analyzed timepoints, accompanied by an increase in cell size starting from 30 h post application (Fig. 7, BLM row). Similar though even more pronounced symptoms were observed upon application of Dd-BLM conjugate, delivering 100 times less BLM (Fig. 7, Dd-BLM).

## Discussion

Dodecahedra composed of twelve Ad3 pentons are produced in human cells upon Ad3 infection [1]. They are synthesized abundantly in Ad3-infected cells in culture, resulting in 5.5×10^6^ Dds produced per one infectious virus [18]. Ad Dd is significantly smaller than the original virus. This is unlike some other known VLPs of non-enveloped viruses such as human papilloma virus (HPV) or Norwalk (Caliciviridae) VLPs, whose VLPs morphologically mimic native virions [19], [20]. In contrast, Ad base dodecahedron, composed of pentameric penton bases normally capping the vertices of the icosahedral virion, is significantly smaller than the complete virus, measuring 280 Å versus approximately 900–1000 Å for the fiberless virions [21]. Lacking several structural components of virions this type of VLP exhibits functional and structural properties different from those of the virus capsid. For example, we have shown that when both primary and secondary receptors are blocked, Ad still infects the cells, but now through interaction of the major virion protein, the hexon, with plasma membrane lipids [22]. Dodecahedra, which do not contain Ad hexons, will be not able to employ this entry mechanism. In contrast, Dd is able to attach to and penetrate cells through interaction with heparan sulfate, a pathway not used by the virus of origin, Ad3 [3], [23]. Since heparan sulfates recognize patches of basic amino acids, this gain-of-function most probably stems from decreased distances between groups of basic residues of neighbouring penton bases in Dd, versus more distant, separated by hexons, penton bases in Ad virions.

Through expression in the baculovirus system we are able to produce DFs and also Dds built of bases alone (DB). Both kinds of Dds very efficiently penetrate cells in culture [2], [4]. Dds assemble in the absence of any viral scaffolding proteins and do not require nucleic acid for assembling. When these dodecahedral particles are isolated from insect cells, they are contaminated with host (insect) cell nucleic acid. The latter can be removed on an ion-exchange column without loss of particle integrity, suggesting its external attachment. This is different, for example, from VLPs of human BK polyomavirus, which seem to pack DNA internally upon assembly [24].

The yield of recombinant Dd production in the baculovirus system is about 10 mg from 100 ml (8×10^7^) of cultured insect cells, which is comparable with a very efficient bacterial system [25]. The purification protocol consists of a simple two-step procedure, yielding non-tagged Dds. However, the particles in the absence of salt have a tendency to aggregate, similarly to papillomavirus VLPs [26]. Ad2 virions have been observed to be rather stable under mildly acidic conditions [6]. In contrast, DB seems to be more stable in the pH range 7 to 8, with about 10°C difference in T_m_ between pH 5 and 7 (Fig. 3), similarly as described for the VLPs of papilloma virus type 11 and of polyoma virus [10], [27].

Our physicochemical and functional analyses have shown that Ad3 Dds exhibit remarkable stability. These particles are stable for long periods of storage at 4°C and even at room temperature. In the presence of 150 mM NaCl they can be stored at temperatures up to 40°C at pH 4–5 and up to 50°C at pH 6–8. Moreover, at increased ionic strength they withstand temperatures up to 60°C (Fig. 3C). Dodecahedra can be frozen and lyophilized in the presence of cryoprotectants, without losing their integrity upon thawing and reconstitution with water. Finally, Dds retain their particulate integrity during incubation in human serum at 37°C for at least 2 h (Fig. 4B). These data show that Dds can be conveniently stored and transported, and can potentially be used for therapeutic purposes under various climates.

We have taken advantage of the remarkable penetration properties of Dds to improve cell delivery of the anticancer antibiotic BLM. Since their discovery, bleomycins have been used in a number of combination anticancer regimens. They are interesting therapeutics as they exert selective tumor cytotoxicity and exhibit low myelo- and immunosuppression [16], [28]. However, despite the fact that as few as 500 BLM molecules introduced into the cytosol are able to kill the cell [12], high BLM doses have to be used in clinical treatment. BLM not only crosses poorly plasma membranes which have very few receptors for BLM [29], but in addition, cell-internalized BLM is inactivated in the cytosol by neutral cysteine aminopeptidase called bleomycin hydrolase [30]. Hence, only a fraction of applied BLM reaches the cell nucleus where it cleaves the DNA [31]. As a result of the high-dose treatment requirements, BLM utility is limited by the systemic toxicity and the dose-dependent pneumonitis, able to progress to lung fibrosis. The latter can affect nearly 50% of total patient population, out of which 3% will die [32]. It is therefore important to be able to decrease the effective dose of BLM.

Dd on its own is only weakly cytotoxic and the cytotoxicity is observed only after prolonged treatment (50 h). Since the largest cytotoxic effect for the Dd-BLM conjugate is seen after the first 10 h of treatment, Fig. 5C), the weak Dd toxicity will not be important in Dd-BLM applications. Moreover, results presented in Fig. 6 show rather normal appearance of Dd-treated cells (row Dd), even at 30 and 50 h.

Ad dodecahedron, similarly to other nanoparticles, possesses a large surface area which can be exploited for multiple ligand presentation. Clustering the ligands on the VLP surface allows multiple ligand-receptor interaction or multivalency. The collective binding between multiple ligands and receptors results in increased affinity. This is even more true for the conjugate of Dd-BLM since not only does one Dd particle bring multiple copies of BLM but, in addition, several types of receptors are in play. Dd recognizes two types of receptors. It has an affinity for the omnipresent heparan sulfates [3], [23], and in this respect it is not very specific for tumor cells (with the possible exception of hepatic cancers). In addition, Dd retains the affinity of its building blocks, penton bases, for av integrins [33]. These integrins are highly expressed on activated endothelial cells and tumor cells but are not present in resting endothelial cells and most normal organ systems; in particular their expression on the neoplastic blood vessels is known to be upgraded [34], [35]. Affinity for av integrins suggests tumor-tropicity of Dd. In addition the Dd-BLM conjugate will be able to recognize the third type of receptors, specific for bleomycin [29]. Thus, BLM-Dd conjugate represents a case of a monovalent ligand attached to the polyvalent carrier, together able to recognize the three cellular receptors.

BLM is a multifunctional molecule composed of three active parts. It was thus possible that its function would be impaired by the cross-linking reaction. However, DLS analysis of Dd-BLM conjugate suggested that the chemical treatment did not introduce gross changes in Dd structure. Application of the Dd-BLM conjugate resulted in cell death, further suggesting that neither the transducing properties of Dd nor BLM cytotoxicity were compromised by the cross-linking reaction. Importantly, the specific DNA-damaging activity of free BLM resulting in dsDNA breaks was retained also by BLM delivered with the dodecahedric vector. Moreover, the bioavailability of BLM delivered with the aid of Dd was 100 times higher than that of free BLM. This is an important result because it shows that BLM delivery can be significantly improved and its cytotoxic effect can possibly be attenuated by attaching it to a suitable vector, conceivably resulting in diminished side effects of BLM therapy. For comparison, when bleomycin was delivered by electroporation, the bioavailability was improved only 2 to 10-fold [36], [37], whereas the use of bleomycin-loaded microspheres did not improve the drug bioavailability when compared to free BLM [38]. Ultrasound application improved BLM bioavailability 8-fold, and the effect was enhanced 33 times when microbubbles were included in the treatment [39]. Finally, the use of liposomes for BLM delivery brought about 20 to 40-fold increase in bioavailability [40], resulting in a significant decrease in BLM-induced lung injury [41].

Induction of giant cells upon Dd-BLM treatment is due to the presence of the antibiotic since cells treated with Dd alone did not exhibit such a phenotype (Figs. 6 and 7). This phenotype resembles the one observed for cells treated with other cell-cycle arresting chemotherapy drugs, where response to treatment quite often results in induction of polyploid giant cells. Drugs interacting with DNA activate several signal transduction pathways, which culminate in the induction of apoptosis. Mitotic or reproductive death after G2 stage arrest is induced by DNA damaging agents and produces enlarged cells containing multiple nuclear fragments, readily distinguished from apoptotic nuclear fragments. After a prolonged incubation, multinucleated cells lose attachment to the tissue culture plate and undergo apoptosis [42], [43]. This has been shown for both transformed cells treated with cisplatin, doxorubicin or etoposide and for established tumors treated with cisplatin [44]–[46].

Different physicochemical approaches such as photochemical internalization, electroporation and ultrasounds, as well as the use of liposomes have been attempted to improve BLM cell entry, its release from endosomes and passage through the cytoplasm [41], [47]–[49]. We propose attaching BLM to a vector that carries out all these functions, namely efficiently penetrates the plasma membrane, is quickly liberated in the cytoplasm and has a propensity to concentrate around the nucleus [2]. Our data on the kinetics of Dd inside transduced cells suggest that the intracellular vector undergoes slow proteolysis, liberating BLM peptides. These small BML conjugates are probably able to easily translocate to the nucleus, where BLM exerts its cytotoxic effect.

It should not be forgotten, however, that we describe here a protein-based system, and therefore the host immune response might pose a major problem in medical application. On the other hand, we wish to apply the Dd-BLM conjugate locally in the glioblastoma animal model, and the nervous tissues are thought to enjoy a conditionally privileged immune status: they are normally unreachable for self-reactive T and B cells, lack lymphatic drainage and are deficient in local antigen-presenting cells. Moreover, it has long been considered that administration of foreign antigens into the brain can lead to a state of tolerance rather than immunization (see [50] and references therein).

In conclusion, it is possible to deliver BLM with the aid of a VLP, Ad Dd, in order to significantly improve drug bioavailability. These experiments open new vistas for improved delivery of impermeant labile drugs, bringing us closer to *in vivo* use of adenovirus dodecahedron as a delivery vector.

## Materials and Methods

### Cells

HeLa cells were cultured in EMEM (Lonza, Basel, Switzerland) supplemented with 10% fetal calf serum (FBS) (Invitrogen, Carlsbad CA, USA), penicillin (50 IU/ml), and streptomycin (50 µg/ml) (Invitrogen) at 37°C, in 5% CO_2_ atmosphere.

### Protein electrophoresis, antibodies, and immunological analyses

Proteins were separated on SDS–PAGE, and stained with Coomassie Brilliant Blue (CBB) or analyzed by Western blotting. The assembly status of purified Dds was analyzed by native agarose gel electrophoresis. Protein samples were mixed with loading buffer (3 mM Tris-HCl, pH 8.0, containing 6 mM NH_4_Cl, 3 mM magnesium acetate, 14 mM potassium acetate, 10% glycerol and 0.005% bromophenol blue) and subjected to electrophoresis in 0.8% agarose gels containing 50 mM Tris and 200 mM glycine, pH 8 at 75 V at 4°C [51]. After electrophoresis proteins were stained with Coommassie Brilliant Blue (CBB) or blotted onto Immobilon-P transfer membrane in 20 mM Tris buffer pH 7.5, containing 150 mM NaCl and 2 mM EDTA.

For Western blot analysis, the rabbit anti-Ad3 Dd (prepared in the laboratory) at 1∶40000 and as the secondary antibody anti-rabbit -horseradish peroxidase (Sigma) at 1∶160000 dilution were used. ECL detection system (Amersham Biosciences, Piscataway NJ, USA) was used throughout this work.

For immunofluorescent microscopy the following antibodies were used: anti-Dd at 1∶1000; anti-tubulin MAb (Sigma, St Louis MO, USA) at 1∶400 and anti-γ-H2AX (polyclonal, Calbiochem, Darmstadt, Germany) at 1∶100 dilution. The secondary Abs were: : goat anti-rabbit FITC-labeled (Santa Cruz Biotechnology, Santa Cruz CA, USA) (1∶200; 1 h at room temperature), sheep anti-rabbit Texas Red-labeled (Jackson, ImmunoResearch Laboratories, West Grove PA, USA 1∶250; 1 h at 37°C), FITC-conjugated goat anti-mouse (Jackson, 1∶250; 1 h at 37°C).

### Dd expression and purification

Full-length human Ad3 penton base gene was cloned and expressed in the baculovirus system [2]. Virus amplification was performed in monolayers of *Spodoptera frugiperda* (Sf21) cells maintained in TC100 medium supplemented with 5% (v/v) fetal calf serum (both from Invitrogen). For Dd expression *Trichoplusia ni* (High-Five, HF) cells, grown in suspension, in Express Five SFM medium (Invitrogen) with gentamycin (50 mg/l) and amphotericin B (0.25 mg/l), were infected with the recombinant baculovirus at multiplicity of infection of 4 pfu/cell. After 48 h cells were collected and lysed by three rounds of freezing and thawing. Clarified lysates were fractionated on 15–40% sucrose density gradients as previously described [2]. Gradients were analyzed by SDS-PAGE with CBB staining.

Heavy sucrose density gradient fractions containing DB were pooled, dialyzed against 20 mM Tris, pH 7.5, containing 2 mM EDTA, 5% glycerol and protease inhibitors (Roche, Indianapolis IN, USA), and subjected to ion-exchange chromatography on a Q-Sepharose column (2 or 5-ml Econo-Pac High Q Cartridge, Bio-Rad, Hercules CA, USA) equilibrated with dialysis buffer. Proteins were eluted at 4°C with NaCl gradient in dialysis buffer. Purified proteins were stored at 4°C in the purification buffer (dialysis buffer containing 280 or 370 mM NaCl). Peak fractions were pooled, and when necessary, concentrated by ultrafiltration in a Microcon unit (cutoff 10000, Millipore, Billerica MA, USA). The purity of protein preparations was assessed by 12% SDS-PAGE. The assembly status of purified recombinant penton base protein was analyzed on native agarose gels and by negative stain EM.

### N-terminal amino acid sequence determination

Aliquots of purified proteins were subjected to SDS-PAGE and electrotransferred onto Immobilon-P PVDF membrane. Amino acid determination based on Edman degradation was performed using an Applied Biosystems gas-phase sequencer model 492 (s/n: 9510287J). Phenylthiohydantoin amino acid derivatives generated at each sequencing cycle were identified and quantified online with an Applied Biosystems Model 140C HPLC system using the data analysis system for Applied Biosystems Model 610A (software version 2.1). The PTH-amino acid standard kit (Perkin-Elmer, Waltham MA, USA) was used and reconstituted according to the manufacturer's instructions. The reagents used to identify and quantify the derivatized amino acids were removed at each sequencing cycle. Retention times and integration values of peaks were compared to the chromatographic profile obtained for a standard mixture of derivatized amino acids.

### Thermal stability studies

Samples of purified Dds were dialyzed at 4°C for 24 h against buffers of different pH and ionic strengths, with 3 changes of each. The samples were stored at 4°C or were subjected to different temperature treatments by incubation in water bath. Some samples were centrifuged for 30 min at 13000 rpm in the Eppendorf centrifuge. The dialyzed proteins and those in the supernatants recovered after centrifugation were analyzed on non-denaturing agarose gels.

Denaturation curves were obtained for Dd and Dd-BLM conjugate as a function of temperature and pH, by the DLS technique. Protein samples (0.2 µg/µl) were dialyzed against pre-filtered (0.45 µm-pore-size filter) buffer solutions. Samples were placed in a reduced-volume cuvette (45 µl, Greiner, Frickenhausen, Germany). Automated measurements were collected with a Zetasizer Nano ZS (Malvern, Worcestershire, UK), using a 2°C incremental temperature ramp, from 12 to 65°C, and a 2 min equilibrium time at each measurement temperature. The data were adjusted using the cumulant method.

### Kinetics of Dd penetration

HeLa cells were attached to the wells of 96-wells plastic dishes (2×10^4^ cells). The medium was removed, the purified Dds (4 µg/100 µl, 10.8 nM) were applied to cells in EMEM medium without FBS, and the dishes were returned to the incubator. Three hours after Dds application the medium was enriched with FBS to 10% final concentration. Cells were collected at the indicated periods (1 to 96 h, see Fig. 4), lysed in Laemmli solution or suspended in hypotonic buffer. Samples containing half of cells in each well were run on SDS-PAGE or native agarose gels, and analyzed by Western blot using anti-Dd antibody.

### Electron microscopy

Samples at approximately 0.1 mg protein/ml were applied to the clean side of carbon on mica (carbon/mica interface) and negatively stained with 1% sodium silicotungstate, pH 7.0. Micrographs were taken under low-dose conditions with a Jeol 1200 EX II microscope (Tokyo, Japan) at 100 kV and a nominal magnification of 40000.

### Bleomycin cross-linking to Dd

Bleomycin A_5_ hydrochloride (Hangzhou Xiangyuan Co., Ltd., China) was chemically cross-linked to purified Dd during a two step conjugation procedure using 1-ethyl-(3-dimethylaminopropyl) carbodiimide hydrochloride (EDC) (Pierce, Rockford IL, USA) and *N*-hydroxysulfosuccinimide (sulfo-NHS) (Pierce). Dd (27 nM) was activated in 0.1 M MES buffer pH 6.0, 0.5 M NaCl, in the presence of 0.31 mM EDC and 5 mM Sulfo-NHS. Cross-linking with bleomycin A_5_ (23 mM) was performed during 2 h incubation with mixing at room temperature. After quenching the reaction by addition of hydroxylamine to a final concentration of 10 mM, chemical reagents and free bleomycin were removed from the preparation by dialysis (24 h, four changes of 20 mM Tris pH 7,5, 150 mM NaCl, 5% glycerol). The amount of bleomycin cross-linked to Dd was evaluated by ms analysis.

### Matrix-assisted Laser Desorption Ionization Time-of-flight (MALDI-TOF) Mass Spectrometry Analysis

Laser desorption/ionization mass spectrometric analysis was performed with a Perseptive Biosystems (Framingham, MA) Voyager EliteXL time-of-flight mass spectrometer with delayed extraction, operating with a pulsed nitrogen laser at 337 nm. Positive ion mass spectra were acquired using a linear, delayed extraction mode with an accelerating potential of 25 kV, a 93% grid potential, a 0.2% guide wire voltage, and a delay time of 1000 ns. Each spectrum represents the results from 100 averaged laser pulses. Samples were concentrated with ZipTipC4 (Millipore) as described by the manufacturer using a saturated solution of sinapinic acid (Fluka) prepared in an 80% (v/v) of acetonitril/0.3% trifluoroacetic acid for elution. The elution mixture was placed on a stainless steel plate and air-dried prior to analysis. External calibration was performed with bovine albumin (Applied Biosystems) using the m/z value of 66431 Da for the mono-charged ion and 33216 Da for the di-charged ion. The values expressed are average mass and correspond to the [M+H]^+^ ion.

### Bleomycin cytotoxicity

Cytotoxic activity of Dd-BLM preparation was tested *in vitro* using MTT (3-(4,5-dimethylthiazol-2-yl)2,5-diphenyltetrazolium bromide) assay, based on the ability of viable cells to reduce a soluble yellow tetrazolium salt (MTT) to blue formazan crystals. HeLa cells grown in a 96-multiwell plate at 10^4^ cells per well were incubated for 3 h at 37°C in 100 µl of EMEM medium without FBS containing different amounts of Dd (where 1 µg amounts to 2.7 nM), BLM-Dd (where 1 µg amounts to 2.7 nM of Dd and 0.08 µM of BLM) or free bleomycin (0.13, 1 and 8 µM). Then FBS was added to a final concentration of 10%. After various times of incubation at 37°C the culture medium was removed and 100 µl EMEM medium containing 0.5 mg/ml MTT (Sigma) was added to each well. The plates were incubated, developed and read according to manufacturer's instructions, in Synergy HTi plate reader (Biotek, Winooski VT, USA ). The number of viable cells was calculated as described by Mosmann [52].

### Confocal microscopy

HeLa cells (5×10^4^) were grown overnight on coverslips. Different amounts of Dd, Dd-BLM conjugate or free bleomycin were applied on cells in EMEM without serum. After 3 h incubation FBS was added to a final concentration of 10% and cells were grown at 37°C. At indicated time points cells were rinsed with cold PBS and fixed in 100% cold methanol for 10 min. Fixed cells were incubated for 1 h with the following primary Abs: rabbit polyclonal anti-Dd, at room temperature, anti β-tubulin MAb (Sigma) at 37°C and rabbit polyclonal anti γ-H2AX (Calbiochem) at 37°C, rinsed with PBS and incubated with the secondary Abs: goat anti-rabbit Texas Red-labeled (Jackson), FITC-conjugated goat anti-mouse (Jackson), and finally with DAPI (Applichem, 1 µg/µl solution, 5 min RT). Antibodies dilutions are given earlier. Images were collected with EZ-C1 Nikon CLSM attached to a inverted microscope Eclipse TE2000 E (Nikon) using oil immersion objective ×60, Plan Apo 1.4 NA (Nikon). DAPI, FITC and Texas Red fluorescence was excited at 408, 488 and 543 nm, and emission was measured at 430–465, 500–530 and 565–640 nm, respectively. Images show a single confocal scans averaged 4 times with 10 µs pixel dwell. All images were collected with 512×512 resolution and zoom 2.0. Figures were processed with EZ-C1 Viewer and Photoshop 6.0.

### FACScan analysis

Non-synchronized HeLa cells were treated with different amounts of purified Dd in EMEM without serum. After 1 h incubation the floating cells were recovered and combined with attached cells harvested by treatment with 2 mM EDTA in PBS. Pooled cells were rinsed with cold PBS and fixed in 100% cold methanol overnight. Fixed cells were pelleted, resuspended in PBS and incubated with the primary anti-Dd antibody (1∶1000; 1 h at room temperature), washed with PBS and then incubated with goat anti-rabbit secondary FITC-labeled antibody (Santa Cruz) (1∶200; 1 h at room temperature). After several PBS washes, portions of approximately 10000 cells were analyzed by flow cytometry on a FACSCalibur (Beckton Dickinson).
